# Oral Health Policies to Tackle the Burden of Early Childhood Caries: A Review of 14 Countries/Regions

**DOI:** 10.3389/froh.2021.670154

**Published:** 2021-06-09

**Authors:** Jieyi Chen, Duangporn Duangthip, Sherry Shiqian Gao, Fang Huang, Robert Anthonappa, Branca Heloisa Oliveira, Bathsheba Turton, Callum Durward, Maha El Tantawi, Dina Attia, Masahiro Heima, Murugan Satta Muthu, Diah Ayu Maharani, Morenik Oluwatoyin Folayan, Prathip Phantumvanit, Thanya Sitthisettapong, Nicola Innes, Yasmi O. Crystal, Francisco Ramos-Gomez, Aida Carolina Medina, Edward Chin Man Lo, Chun Hung Chu

**Affiliations:** ^1^Guanghua School of Stomatology, Hospital of Stomatology, Sun Yat-sen University, Guangzhou, China; ^2^Faculty of Dentistry, The University of Hong Kong, Hong Kong, China; ^3^Faculty of Health and Medical Sciences, University of Western Australia, Perth, WA, Australia; ^4^School of Dentistry, Rio de Janeiro State University, Rio de Janeiro, Brazil; ^5^Faculty of Dentistry, University of Puthisastra, Phnom Penh, Cambodia; ^6^Faculty of Dentistry, Alexandria University, Alexandria, Egypt; ^7^Faculty of Dentistry, Kagoshima University, Kagoshima, Japan; ^8^Centre for Early Childhood Caries Research (CECCRe), Sri Ramachandra Institute of Higher Education and Research, Chennai, India; ^9^Adjunct Research Associate, Centre of Medical and Bio-Allied Health Sciences Research, Ajman University, Ajman, United Arab Emirates; ^10^Faculty of Dentistry, University of Indonesia, Jakarta, Indonesia; ^11^Faculty of Dentistry, Obafemi Awolowo University, Ife, Nigeria; ^12^Faculty of Dentistry, Thammasat University, Khlong Nueng, Thailand; ^13^School of Dentistry, Cardiff University, Cardiff, United Kingdom; ^14^College of Dentistry, New York University, New York, NY, United States; ^15^School of Dentistry, University of California, Los Angeles, Los Angeles, CA, United States; ^16^School of Dentistry, Universidad Central de Venezuela, Caracas, Venezuela

**Keywords:** child, dental caries, early childhood caries, fluorides, policy, oral health

## Abstract

**Aim:** Early childhood caries (ECC) has significant public health implications but has received inadequate global attention. There is limited information regarding the success of oral health policies implemented to address the challenges of ECC. This review aimed to summarize such policies to tackle ECC from different countries/regions.

**Method:** Independent collaborators from 14 countries/regions (Australia, Brazil, Cambodia, China, Hong Kong, Egypt, India, Indonesia, Japan, Nigeria, Thailand, UK, USA, and Venezuela) collected the data. The ECC status, dental workforce, oral health policies on ECC prevention in different countries/regions were summarized by each country.

**Results:** The findings indicated that ECC prevalence varied in different countries/regions. The lowest prevalence of ECC among 5-year-old children was found in Nigeria (7%), and the highest was found in Indonesia (90%). The existing dental workforce and resources are limited in most countries. The smallest dentist to population ratio was reported by Nigeria at 1:48,400, whereas the highest ratio was in Brazil (1:600). Out of 14, three (21%) countries namely India, Venezuela and Cambodia had no national oral health policies addressing ECC and four (29%) countries (Cambodia, China, India, Venezuela) had no publicly funded dental care program for 0–5-year-old children. Water fluoridation is available in four countries/regions (Australia, Brazil, Hong Kong, USA).

**Conclusion:** ECC remains a global health challenge and dental workforce is limited. National/regional programs to tackle ECC are not yet prioritized in many countries/regions. Evidence to support demonstration projects is limited. Further research on the cost-effectiveness of interventions strategies is required for policymakers.

## Introduction

Early childhood caries (ECC) is defined as the presence of at least one decayed (cavitated or non-cavitated), filled or missing (due to caries) tooth in children younger than 6-years of age [1]. The prevalence of ECC remains high and has significant impacts on children's oral health, general health and well-being throughout their life course [2]. The Global Burden of Disease study in 2017 reported that approximately 530 million children had untreated caries in their primary teeth [3]. When untreated caries progresses, ECC can compromise the ability to chew and eat, limit daily activities, and negatively affect children's growth and development [4]. ECC also has significant impacts on children's families and societies, including carrying a financial burden, because tertiary treatment of ECC under sedation or general anesthesia is costly and unaffordable for many families [5].

ECC remains a global issue [6]. A number of risk factors are associated with ECC. Individual-level risk factors for ECC include behavioral factors such as feeding patterns and oral hygiene habits [7]. Family-level factors, such as socio-economic aspects, are associated with ECC [8]. Community-level factors such as environment, poverty, maternal well-being, dental care access and universal dental coverage are also related to ECC prevalence [9]. Research has also demonstrated that ECC is associated with other common childhood diseases, and shares similar risk factors with other non-communicable diseases [10].

The burden of ECC is enormous and requires urgent action to improve children's oral health globally [11]. Government policies have direct influence on the provision of oral health care to populations and can also moderate a range of structural risk factors for ECC. Although some recent studies investigated the efficacy of new anti-caries agents (i.e., silver diamine fluoride (SDF) solution) for arresting dentine caries or new intervention protocols on ECC [12, 13], there are few studies investigating how research findings have been translated into practice, and how policymakers and general dental practitioners have responded to new guidelines on ECC management such as guidelines developed by the American Academy of Pediatric Dentistry [14, 15]. Every country has implemented their own strategies to tackle ECC [11]. Sharing experiences from community settings across different countries would assist in building a body of evidence around which policies work, which do not work, and under what circumstances. This would inform which strategies might be best to be adopted at a national level by other countries. Such information would be beneficial for health policymakers, dental educators, clinicians and researchers to allow reformulation of their current oral health policies, training curricula and research directions for ECC to be based on evidence. This would increase the chance of them translating into benefit for the child populations in their communities and countries. This review aims to describe current oral health policies for tackling the burden of early childhood caries in 14 countries/regions.

## Method

Twenty-two researchers from 14 countries or regions, namely Australia, Brazil, Cambodia, China, Hong Kong, Egypt, India, Indonesia, Japan, Nigeria, Thailand, United Kingdom (UK), United States of America (USA), and Venezuela collaborated on this review. Independent collaborators in each country were asked to collect specific data points including ECC status, dental workforce and oral health policies related to ECC for their own countries. These data were presented in tables. Collaborators also developed a country-level narrative to discuss their perspectives on the lessons learned, challenges and barriers to ECC management, and large-scale or national oral health programs in their own countries independently. All collaborators in each country retrieved the information independently from published articles, government documents, official websites and personal communication with government authorities.

## Results

**Figure 1:**
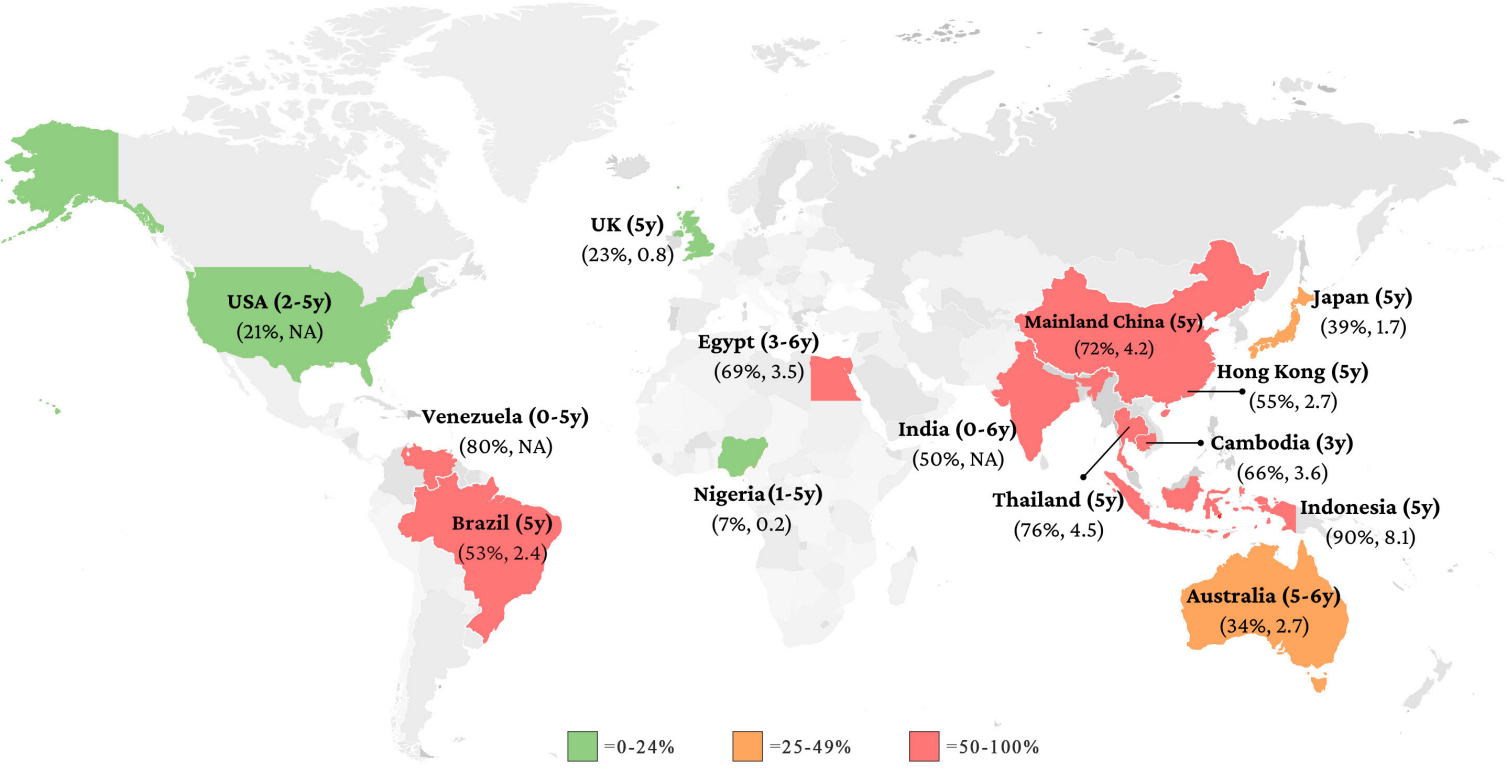
Caries prevalence (%) and caries experience [decayed, missing and filled teeth (dmft) index] of primary teeth in children.

The information of ECC status was summarized in Table 1 and a global map on ECC status was developed accordingly (Figure 1). The findings indicate that ECC prevalence varied between countries/regions. The lowest prevalence of ECC among 5-year-old children was found in Nigeria (7%) and the highest in Indonesia (90%).

**Table 1:** Early childhood caries: prevalence and caries experience in different countries/regions included in this review.

**Country/Region**	**Year of data collection**	**Sample size**	**Caries prevalence**	**dmft Mean (SD)**
Australia [16]^#^	2016	5,130	5–6 y: 34%	2.7
Brazil [17]^#^	2010	7,217	5 y: 53 %	2.43
Cambodia [18]	2017	3,985	1 y: 20% 2 y: 45% 3 y: 66%	1 y: 0.7 (1.7) 2 y: 2.0 (3.1) 3 y: 3.6 (4.1)
China [19]^#^	2015	40,360	3 y: 51% 4 y: 64% 5 y: 72%	3 y: 2.3 (3.4) 4 y: 3.4 (4.2) 5 y: 4.2 (4.5)
Egypt [20]^#^	2013–2014	651	3–6 y: 69%	3–6 y: 3.5 (3.6)
Hong Kong [21]^#^	2016	1,204	3 y: 38% 4 y: 43% 5 y: 55%	3 y: 1.4 (2.9) 4 y: 1.9 (3.2) 5 y: 2.7 (3.8)
India [22]	2019	–	0–6 y: 50%^*^	–
Indonesia [23]^#^	2018	1,872	3–4 y: 81% 5 y: 90%	3–4 y: 6.2 5 y: 8.1
Japan [24]^#^	2016	165	1 y: 0% 2 y: 7% 3 y: 9% 4 y: 36% 5 y: 39%	1 y: 0^***^ 2 y: 0.3 (1.1)^***^ 3 y: 0.3 (1.2)^***^ 4 y: 0.9 (1.7)^***^ 5 y: 1.7 (3.1)^***^
Nigeria [25]	2014	497	1–5 y: 7% 1 y: 2% 2 y: 2% 3 y: 8% 4 y: 13% 5 y: 7%	Overall: 0.15 1 y: 0.13 2 y: 0.02 3 y: 0.14 4 y: 0.27 5 y: 0.21
Thailand [26]^#^	2017	8,308	3 y: 53% 5 y: 76%	3 y: 2.8 5 y: 4.5
UK [27]^#^	2019	6,900	5 y: 23%^**^	5 y: 0.8
USA [28]^#^	2016	7,000	2–5 y: 21%	–
Venezuela [29]	2013–2014	293	0–5 y: 80%	–

*dmft, decayed, missing and filled teeth*.

* *The data are from a systematic review not a population-based data*.

** *No reliable combined data for all UK (i.e., England, Scotland, Wales, and Northern Ireland). Data for England are the most representative, although may be a slight underestimate*.

*** *Based on dft score instead of dmft score*.

# *Data were extracted from national/territory-wide survey*.

The information on the dental health workforce, including the number of dentists, pediatric dentists, and the ratio of dentists to population in each country/region which is shown in Table 2. The dental workforce and resources are limited in most countries. The smallest dentist to population ratio was in Nigeria at 1:48,400, whereas the highest ratio was in Brazil (1:600). ECC management is not a priority in most countries. Current oral health policies on ECC in each country are described below and summarized in Table 3. Out of 14, three (21.4%) countries, namely India, Venezuela and Cambodia, had no national oral health policies targeting ECC and four (28.6%) countries (Cambodia, China, India, Venezuela) had no publicly funded oral health programs for 0–5-year-old children. Fluoride toothpaste is commercially available in all countries. Water fluoridation is available in four countries/regions (Australia, Brazil, Hong Kong, USA). SDF is available in the market in most countries/regions including Australia, Brazil, Cambodia, Egypt, Hong Kong, Indonesia, India, Japan, Thailand, UK, and USA while it is not available in China, Nigeria and Venezuela. Government-subsidized primary prevention (dental screening and fluoride treatment, etc.) for young children provided by primary healthcare providers is available in countries including Brazil, Indonesia, Nigeria, Thailand, UK, and USA while it is not available in Australia, Cambodia, China, Egypt, India, Japan, Hong Kong, and Venezuela. A country-level narrative on the lessons learned, challenges and barriers to ECC management, and large-scale or national oral health programs are discussed as follows.

**Table 2:** Updated dental health workforce data: number of dentists, pediatric dentists, ratio of dentist to population, and ratio of pediatric dentist to child population (aged 0–5-years).

**Country/Region**	**Overall population (1,000,000) (a)**	**No. of children aged 0–5-years (1,000,000) (b)**	**No. of dentists (c)**	**No. of registered pediatric dentists (d)**	**Ratio of Dentist: Overall population (c/a)**	**Ratio of Pediatric dentist: Children aged 0–5-years(d/b)**
Australia	25.5 [30]	1.6 [30]	21,838 [30]	156 [30]	1:1,100	1:10,300
Brazil	211.8 [31]	14.7 [31]	342,193 [32]	8,953 [33]	1:600	1:1,600
Cambodia	16.9 [34]	1.7^*^ [35]	1,826 [36]	2^#^	1:9,300	1:850,000^#^
China	1,400.0 [37]	90.3 [37]	245,000 [38]	–	1:5,700	–
Egypt	102.7 [39]	15.0 [39]	19,111 [40]	–	1:5,400	–
Hong Kong	7.5 [41]	0.3^*^ [41]	2,381 [42]	38 [43]	1:3,100	1:7,900^*^
India	1,300.0 [44]	163.8^**^ [44]	277,503 [45]	4,000 [45]	1: 41,100	1: 41,000^**^
Indonesia	262.0 [46]	23.7 [47]	31,654 [48]	522 [48]	1:8,300	1:45,400
Japan	125.9 [49]	5.9 [49]	104,533 [50]	–	1:1,200	–
Nigeria	140.4 [51]	27.1 [51]	2,901 [52]	–	1:48,400	–
Thailand	66.6 [53]	4.0 [53]	15,951 [54]	527 [55]	1:4,200	1:7,600
UK	67.9 [56]	4.9 [57]	35,000 [58]	246 [59]	1:1,900	1:19,910
USA	330.0 [60]	23.2 [61]	200,500 [62]	8,000 [63]	1:1,600	1:2,900
Venezuela	27.2 [64]	2.4^*^ [64]	17,000 [65]	93 [65]	1:1,600	1:25,800^*^

* *0–4-year-old children*,

** *0–6-year-old children*,

# *Personal Communication: Email correspondence between Dr. Bathsheba Turton (Technical adviser) and Director of Oral Health Bureau, Ministry of Health, Cambodia, 23 September 2020*.

**Table 3:** Updated oral health policies related to ECC in each country/region.

**Country**	**National oral health policy/program to control ECC**	**Preventive measures**	**Public dental healthcare insurance for children aged 0–5**
Australia	Australia's National Oral Health Plan 2015–2024	– Water fluoridation	– For children from lower income families
Brazil	Smiling Brazil National Oral Health Program (NOHP) since 2004	– Water fluoridation – Individual educational-preventive actions with pregnant women – Supplying free toothbrushes and fluoride toothpaste	– Universal coverage
Cambodia	No national oral health program	– No national preventive measures	– No coverage
China	The National Program for Chronic Disease Control and Prevention (2017–2025), an action plan for the Healthy China 2030	– Promote oral health education in kindergartens	– No coverage
Egypt	Social Health Insurance Law of 2018 to be fully implemented all over Egypt in 15-years offering Social Health Insurance Program (SHIP)	– Basic oral health package (health education with or without fluoride application) proposed under SHIP following evidence-based guidelines	– Currently, family Health units offer therapeutic care in a fee-for-service model at low prices and provide mostly extractions
Hong Kong	Oral Health Education Unit (OHEU) aims to improve the oral health status of Hong Kong preschool children since 1989	– Water fluoridation with 100% coverage – Provide only oral health education for parents and preschool children	– Only children of civil servants
India	No national oral health program	– No national preventive measures	– No coverage
Indonesia	The National Health Insurance (NHI) scheme 2014	– Primary, secondary, and tertiary oral health care prevention schemes	– Universal coverage
Japan	The 8020 campaign (since 2000)	– Checkups at 6 months, 8 months, 1-year-old, 2-year-old, and an annual dental check-up at their kindergartens fissure sealants on primary molars	– Universal coverage
Nigeria	The 2020 National Oral Health Policy	– Promote access of pregnant women receive routine oral care – Promote children's visit to the dentist by their first birthday – Ensure access and use of fluoridated toothpaste daily by pre-school children	– Universal health coverage
Thailand	National Health Act	– Sugar taxation – Oral health promotion: brushing campaign when the first tooth erupts – Oral health examination and fluoride varnish application at the day care centers	– Universal health coverage including preventive restoration (SMART), simple treatment such as extraction, filling, etc.
UK	Child Dental Health Care under National Health Service (NHS)	– Oral health promotion (Childsmile, Scotland and Designed to Smile, Wales) – Primary, secondary and tertiary intervention – Soft Drinks Industry Levy: reducing sugar content of drinks since 2016	– Universal health coverage under NHS but different payment systems in each nation
USA	Medicaid and Children's Health Insurance Program (CHIP)	– Water fluoridation – Dental home program – Headstart (HS) and Women, Infants and Children (WIC) provide education, screenings, check-ups, nutrition services and referrals	– Comprehensive dental coverage for families below the poverty level
Venezuela	No specific oral health policy	– Salt fluoridation	– No coverage

### National Oral Health Care Plans and Policies for Each Country to Tackle ECC

#### Australia

The National Child Oral Health Study 2012–14, a cross-sectional study of children aged 5–14, provided recent data on child dental health in Australia. Around one-third (34%) of Australian children aged 5–6 had caries experience in their primary teeth [16]. Furthermore, over one-quarter (26%) of Australian children had untreated decay in their primary teeth. In addition, aboriginal and Torres Strait Islander children exhibited a 1.5 to 2.5 times higher prevalence of dental caries experience and untreated dental caries in both primary and permanent teeth compared with the national average [66]. Approximately half of the Australian children have not visited a dentist before their fifth birthday.

Children living in remote and rural areas (53%) exhibit more caries and untreated carious primary teeth than children in major cities (39%). Therefore, it is evident that socio-economic profile and geographical remoteness influence the availability of, and accessibility to dental care [67]. Similarly, fluoridated tap/public water, although provided in most Australian states and territories; its coverage varies across each jurisdiction. Children in high-income households more likely to drink tap water (79%) than children in low-income families (65%) [68].

The infrequent conducting and reporting of national surveys of children's oral health status makes monitoring changes in children's dental health challenging. Although initiatives to enhance dental care access may have an immediate impact on untreated dental caries prevalence, initiatives to prevent dental caries will require a lifetime approach. Currently, National Oral Health Plan 2014–2024 is a blueprint for action across different sectors and jurisdictions to ensure Australian children and adults have healthy teeth and mouths.

#### Brazil

More than 50% of Brazilian children have already experienced dental caries by age 5 and 80% of their carious teeth remained untreated [69]. In the past decades, national efforts to control ECC have included: expansion of community water fluoridation coverage and improvement of its surveillance systems, universal mother and child health care free of charge, and health promotion policies aiming at increasing the use of fluoride toothpastes and decreasing the consumption of sugary products. Fluoridation of tap water within a range of 0.6 to 1.2 ppm concentration is mandatory in Brazil. During the first decade of the twenty first century, water fluoridation coverage was expanded by 8.6% reaching 76.3% of the population. This expansion was more pronounced in municipalities with <10,000 inhabitants and in those with low or very low Human Development Index [70].

Health care is a constitutional right and the creation of the Unified Health System in 1990 marked a shift in the Brazilian model of health care from hospital-centered to community-centered. In 1994, the Family Health Programme (FHP) was implemented and became the core of the system. The FHP multi-professional teams performed health promotion and disease prevention interventions and provided disease management both at home and at primary health care centers, mostly focused on maternal and child services [71]. In 2001, oral health teams (OHT) were incorporated into the FHP. In 2004, the Smiling Brazil National Oral Health Program (NOHP) was launched. It contained a number of proposals aiming at preventing ECC such as: conducting collective and individual educational-preventive actions with pregnant women, creating opportunities for infant's early contact (no later than by 6 months of age) with the OHT (e.g., utilizing vaccination campaigns to introduce good oral health habits) and performing group activities and home visits for identification and referral of children at high risk or with dental needs for individual care [69]. From 2003 to 2009, OHT population coverage increased from 20.5 to 47.5%. However, despite growing numbers of OHT from 2009 to 2017, population coverage fell to 36.7% in 2017 [72]. In addition, not all (81%) of the OHT provided dental care for children younger than five [73].

Supplying free toothbrushes and fluoride toothpaste regularly to children served by the FHP is one of the cornerstones of caries prevention in the NOHP. Nevertheless, regulation regarding fluoride toothpastes marketed in the country establishes the maximum amount of fluoride that they can contain (1,500 ppm) but does not set standards regarding their minimum concentration of soluble fluoride. Therefore, it was commonly found that toothpastes distributed to children by FHP teams had little or no anti-caries benefit [74, 75]. Before 2014, fluoride toothpastes were not recommended for children up to 4-years of age. In 2015, the Children's Health Handbook, which provided information to parents regarding preventive health measures, was updated. Since then, caregivers have been advised to start brushing their children's teeth with a small amount of fluoride toothpaste from the emergence of the first tooth.

Nowadays, high sugar intake is a widespread dietary practice in Brazil; more than 80% of children have been exposed to sugar by 6 months of age [76]. In order to promote healthy eating habits, recently published national dietary guidelines recommend avoiding the consumption of ultra-processed foods and not offering sugary foods or beverages to children up to 2-years of age.

Thus, there remains considerable room for improvement in early childhood oral health care access in Brazil. Monitoring disease trends more closely and evaluating the impact of policy changes regarding the use of fluoride toothpastes and sugar consumption on ECC prevalence may contribute to the adoption of evidence-based targeted measures of disease prevention and control. Moreover, one cannot overlook that social factors are major drivers of dental diseases. Thus, sustainable positive changes in Brazilian children can only be achieved by reducing socioeconomic inequalities and improving people's overall quality of life.

#### Cambodia

The prevalence and severity of ECC in Cambodia had been increasing in the recent decades. The latest survey found that 66% of the 3-year-old children had ECC experience [18] and addressing it was a complex problem [77]. Research had shown that ECC was impacting the growth and development of many children to the extent that Cambodian children with severe ECC had nearly twice the chance of developing stunting malnutrition [78], and one in 10 families reported an impact on their household functioning as a result of their child's oral condition [18]. In recent years, there has been some success in piloting models of care; however, they are yet to attract central government support for an upscale. One model of care for infants tested in Cambodia involved primary prevention (oral health promotion, application of fluoride varnish, delivery of fluoride toothpaste) provided by primary healthcare personnel and integrated with the routine delivery of prenatal and postnatal care (vaccinations, deworming, and vitamin A supplementation). This approach resulted in a 40% reduction in disease for participating children [79]. A second demonstration project delivered secondary and tertiary prevention (glass iononer sealants, restorative work, and SDF) once children reached a preschool or primary school setting [80]. An important piece of delivering these interventions is the use of the mid-level provider, the Cambodian dental nurse, in delivering care at scale through government systems. At present Cambodian dental nurses have very few opportunities to be employed within the primary health care system. Although the results of the demonstration projects have been positive [79], further work is needed to achieve broad stakeholder buy-in, attract central government resources, and integrate these strategies within the primary health care and education systems.

Even if such programs were to be implemented at scale, it would not address the structural drivers of the disease such as policies and systems that compromise the food environment or prevent social mobility. Added to this is the lack of water fluoridation. In the Cambodian context, it is important to avoid a victim-blaming approach that assumes that families do not have knowledge about oral health. Reported data from Cambodia show that most parents do know about fluoride toothpaste and that sugar is a key cause of caries [18]; this suggests that it is probably not a gap in factual knowledge that is driving the extreme disease experience. ECC shares a number of risk factors with other non-communicable diseases. This calls for a common risk factor and multi-disciplinary approach. The challenge is how to use systems thinking to design and implement complex approaches that address the problem of ECC across disciplines, industries, and social structures.

#### China (Mainland)

ECC is a major public health problem in China. Over 70% of the 5-year-old Chinese children suffered from dental caries at the time of the latest national oral health survey [19]. In recent decades, the Chinese government has been searching for safe and effective dental caries prevention strategies to manage this oral disease epidemic.

Water fluoridation was first introduced in Guangzhou City, Guangdong Province with a fluoride concentration level of 0.8 parts per million (ppm) in 1965 [81]. The incidence rate of dental caries decreased by ~20 to 60% in different areas after the implementation of water fluoridation [82]. However, this program ceased in 1983 due to the 6.5-fold increase in dental fluorosis [83]. How to balance the benefits of water fluoridation for ECC prevention and the risk of dental fluorosis continues to attract public attention and arouses a heated discussion in China today. Apart from water fluoridation, the health care system in China provides basic medical coverage for almost all of the population, but the coverage varies in different areas and depends on local regulations. It is estimated that in China over 85% of the total oral care costs is at the patients' own expense [84].

In 2016, the Chinese government was dedicated to improving Chinese people's health and making public health a precondition for future economic and social development. Accordingly, the Healthy China 2030 blueprint and associated action plans were released [85]. The National Program for Chronic Disease Control and Prevention (2017–2025), an action plan for the Healthy China 2030 implementation, proposed several strategies related to oral health promotion, including: (1) promoting oral health education in kindergarten, primary and middle schools; (2) developing oral health-related techniques and instruments; (3) promoting early intervention in community health service centers and township hospitals; (4) integrating oral examination into the regular physical examination; (5) developing a personalized interventions for children and the elderly, which focus on dental caries and periodontal disease management; and (6) providing topical fluoride, pit and fissure sealing and other oral health-care measures to reduce the caries prevalence rate to below 30% by 2025 [86]. Though not all mentioned strategies are targeting preschool children, preschoolers may still benefit from this national policy if the afore mentioned strategies are well-implemented. The actual implementation program should be investigated and their effectiveness on ECC management needs to be confirmed in the future.

#### Egypt

Egypt is the most populous country in the Middle East and North Africa with about 100 million inhabitants, 15 million of whom are younger than 6-years of age and 57% of whom live in rural areas [39]. In the last 10-years, studies conducted mainly in Northern Egypt reported an early childhood caries prevalence of 69.6% in 2013 [87] and 75% in 2014 [88]. A nationally representative study published in 2019 reported high prevalence (69.2%) with a mean dmft (decayed, missing and filled teeth) score of 3.5 among the 3-to 6-year-old children [20].

In Egypt, there is no fluoridation program and there are wide variations in fluoride concentration in natural water. Also, <2.9% of the children aged 5 or younger had ever used fluoride tablets and 1% had received professional fluoride application [89]. The Demographic Health Survey showed that only 13% of mothers exclusively breast-fed their children up to 5 months of age [90] and the introduction of complementary food with high cariogenic potential may have contributed to high ECC prevalence. In addition, the prevalence of daily brushing in 2- to 5-year-old children was reported to be 22.7% [89].

Therapeutic care covering the dental treatment needs of preschool children used to be in the Ministry of Health Mother and Child Health units that were later restructured into Family Health Units. These units offered fee-for-service care at low charges for pregnant women and their children which they paid for out of pocket with no insurance coverage. Most services in Family Health units were emergency such as extraction. A low percentage of <6-year-old children is covered by private insurance schemes, mostly being those whose parents are members of professional unions. Treatment such as pulpotomy constituted 0.4% of all claims and 0.3% of the total cost of care in these private insurance schemes [89].

The new Social Health Insurance Law of 2018 provided universal health care for all citizens in order to provide access to health services without financial hardship. The main features of the law are the separation of health services purchase, provision and regulation in addition to extending coverage to include the family as a unit. The law imposes a payment of 1–4% deducted from the total monthly income which is significantly higher than the current payment. Heads of households will pay the share of family members including 0.5% for each child. Provision of dental care is explicitly stated in the law. Under this law, preschool children will be health-insured so that it would be possible to develop a basic package aiming at the prevention of ECC including health education, dietary counseling, fluoride application and/ or other evidence-based modalities. The enactment of the law will proceed in phases to cover the whole country within 15-years [91]. Assessing methods to deliver timely, cost-effective and culturally tailored services are challenges for the implementation in preschool children with high caries prevalence.

#### Hong Kong

The latest territory-wide survey showed that around half (55%) of the 5-year-old children had ECC and more than 90% of the decayed cavities remained unrestored [92]. The government of Hong Kong has been actively implementing several oral health promotion programs to manage ECC. Water fluoridation was the first territory-wide program, which was launched in 1961 at a level of fluoride concentration of 0.8 parts per million (ppm). In 1988, the fluoride concentration was adjusted to 0.5 ppm [93]. Water fluoridation is considered an important dental public health measure in Hong Kong, with 100% of the population consuming fluoridated water. In 1989, The Department of Health set up the Oral Health Education Unit (OHEU), which aims to improve the oral health status of Hong Kong preschool children [93]. Free oral health educational materials such as leaflets are available for parents and kindergarten teachers. After the implementation of water fluoridation and establishment of the OHEU, the oral health status of preschool children in Hong Kong significantly improved during the 1960s and 1990s [94]. Non-governmental organizations also launched short-term community oral health services, including free oral examination and topical fluoride treatment for preschool children. However, these short-term projects can only benefit a small portion of preschool children in Hong Kong. Nevertheless, the prevalence of ECC has shown no significant change since 2001 [93].

The Faculty of Dentistry at The University of Hong Kong, the only dental school in Hong Kong, had implemented a pilot “Preschool Oral Health Program” in 2008–2009. Later, this program was scaled up and continued providing dental screening and SDF treatment for caries control for kindergarten children from 2010 to 2019. Several clinical studies of SDF were conducted in Hong Kong and all studies showed favorable results of SDF in arresting dentine caries [12, 95]. Recently, the Faculty of Dentistry at the University of Hong Kong launched the “Jockey Club Children Oral Health Project” (JCCOHP) in 2019, which is supported by The Hong Kong Jockey Club Charities Trust [96]. This large-scale school-based oral health project aims to screen all of Hong Kong's preschool children for ECC, control existing cavities with SDF treatment, and prevent dental caries through oral health education for parents and teachers. Nevertheless, there still exist areas for continued improvement. The effectiveness of this large-scale kindergarten-based dental care service in managing ECC needs to be confirmed. Impacts of this program on oral health, as well as societal and economic aspects should be systematically evaluated and documented.

#### India

A systematic review summarized that the prevalence rate of ECC among 0- to 6-year-old children in India was 50% [22]. The Government of India's Ministry of Health and Family Welfare has envisaged the National Oral Health Program for affordable, accessible, and equitable oral health care delivery with a well-coordinated manner of bringing about “optimal oral health” for all [97]. The objectives of this program are to improve the determinants of oral health, reduce morbidity from oral diseases, integrate oral health promotion and preventive services within the general health care system, and encourage the promotion of a public private partnership model for achieving better oral health. To achieve these objectives, the government has decided to assist state governments in initiating the provision of dental care along with other ongoing health programs implemented at various levels of the primary health care system under the umbrella of the national health mission. The Government of India also helps in developing prototype information, education and communication materials/behavior change communication materials for dissemination of information and to raise awareness about oral health across the country.

Rashtriya Bal Swasthya Karyakram (RBSK) is an important initiative under the National Rural Health Mission (NRHM) which aims at early identification, and early intervention for children from birth to 18-years to cover the 4 D's, namely defects at birth, deficiencies, diseases, and development delays including disability [98]. This scheme enables the 0–6-years age group to be specifically managed at District Early Intervention Centers (DEIC). Child health screening and early intervention services under RBSK are envisioned to cover 30 selected health conditions for screening, early detection, and management. Dental conditions are included amongst these conditions under the ambit of the RBSK scheme. Nevertheless, only a few provinces in the country have adapted this and recruited pediatric dentists for the DEIC. Even though dental conditions are one of the D's, specific guidelines on detection of early changes or white spots lesions have not been specifically addressed. In the future, this channel of health care delivery can be used to introduce specific preventive measures to prevent ECC at the population level.

Currently in India, no large-scale primary, secondary, or tertiary-level preventive programs targeted to prevent ECC exist. The recently launched entity named “Centre for Early Childhood Caries Research” (CECCRe) is established as the first dedicated ECC research center in the country. CECCRe primarily focuses on ECC prevention. Presently, a few cost-effective sustained interventions are being tested in small cohorts of cleft children. The preliminary results have been very encouraging in terms of early identification of early changes, thereby preventing ECC by a principle called Sustained Anticipatory Guidance (SAG). CECCRe is planning to integrate the delivery of cost-effective preventive measures through RBSK and NRHM in the coming years. The projects being carried out at CECCRe focus on studying epidemiology, etiology, behavioral factors, host factors namely enamel surface, morphology, contact areas, ECC and general health, and cost-effective interventions to control ECC at both individual and population level.

In India, the formidable challenge is emphasizing oral health as a national priority. In addition, increasing the awareness regarding prevention of ECC among the common man, health care professionals, and dental professionals is a large task. The country has made significant efforts to handle other health care burdens including infant mortality and communicable diseases. However, evidence suggests that the prevention of ECC can improve the general health of infants and children. This may persuade health policymakers to effectively integrate ECC prevention into RBSK and execute through Early Intervention Centers across the country in the near future.

#### Indonesia

The prevalence rate of ECC in Indonesia was reported to be 90% for 5-year-old children in 2018 [23]. Indonesia is a middle-income country with 262 million inhabitants from more than 300 ethnicities and spread over 17,744 islands. The ratio of dentists in 2004 and 2019 was 5 and 13 dentists, respectively, per 100,000 people [46]. A national policy on oral health was published by the Indonesian Ministry of Health decree number 89-Year 2015 and stresses oral health as an integral part of general health. Although primary, secondary and tertiary oral health care prevention was described comprehensively in that decree, unfortunately information regarding its implementation and evaluation is limited. Population measures such as affordable fluoridated toothpaste, oral health education, and parental counseling are available. Nonetheless, these approaches, which include topical fluoride, sealants and SDF programs, are not yet included in a nation-wide program due to limited resources. As one strategy, the Indonesian Government implemented the National Health Insurance scheme, the world's largest social health insurance, which was initiated to improve access to health care for of all citizens including children in remote and disadvantaged communities [23]. The prevalence rate of dental caries is high among Indonesian children due to various factors including frequent sugar consumption. Preventive programs should address sugar restriction, particularly addressing the practice of adding sugar to milk [99]. Mandatory school programs that increase tooth-brushing frequency with fluoride toothpaste to twice daily would be feasible and sustainable to implement. Although access to limited oral health personnel has changed toward equality and equity, socioeconomic, and geographic barriers remain a challenge in access to dental care for young children in Indonesia.

#### Japan

In Japan, there is a comprehensive governmental strategy to improve oral health for all ages, instead of a strategy solely focused on ECC [100]. This strategy includes care for pregnant mothers and children up to 5-years-old. This governmental strategy has been known as the 8020 campaign; the goal is for individuals to keep 20 of their own teeth until the age of 80 to enjoy eating with their own teeth for their entire life. The prevention of ECC is conducted as the start-line of good oral health throughout the life course. This campaign has continued for over 30-years. At its start, only 10% of people 80-years of age had 20 teeth; however, the number had increased to 50% by 2016 [100]. Furthermore, it does not only affect oral health but also helps to improve the overall quality of life [101].

As a governmental strategy, the barrier of dental utilization for children has been minimized. Everyone in Japan has dental insurance, and their out-of-pocket expenses for those in kindergarten and younger are also covered by local governments. Thus, their cost of dental care is zero. As another part of the strategy, well-being checkups at 18 months and 3-years old are conducted as a government project, and in 2017 acceptance rates were 96.2 and 95.2%, respectively [102]. City or other local governments conduct other well-being checkups at other ages, such as 6, 8 months, 1-, 2-years-old, and so on. Furthermore, all students, starting from kindergarten to high school level, have to receive an annual dental check-up at their schools. The school dentists are required to report the children's dental condition to the child's parents and refer them to their dentist as needed. Schools also have to report the attendance rate of dental check-up, oral health information of their pupils, such as dft, and the rate of receiving follow-up dental care to the government.

Government also encourages dentists to conduct preventive care in their dental clinic through a “dental home” program. When a dental clinic is approved as a “dental home” by the Bureau of Health and Welfare, these dental homes will receive incentives from insurance organizations when they enhance preventive care programs.

Regarding the use of fluorides, government and dental professional groups recommend water fluoridation, but local water departments are pessimistic due to the previous fluorosis problems. Toothpastes can contain fluoride up to 1,450 ppm. SDF was applied to children in the 1970–1990s, however, SDF is currently seldom used for caries management in Japan. Regarding dental sealants, they are proactively placed on primary molars and helps to prevent dental caries [103]. In conclusion, oral health problems, including ECC, have decreased in Japan with the contribution of a governmental strategy to reduce the barrier of dental utilization and to encourage dentists toward preventive care.

#### Nigeria

In Nigeria, the prevalence rate of ECC was 7% for 1- to 5-year-old children in 2014 [25]. The goal of the 2020 National oral health policy for Nigeria is to *strengthen the national health system so that it will be able to provide gender-sensitive, equitable, accessible, affordable and efficient oral health services that meet the preventive, curative and rehabilitative health needs of all Nigerians, thereby reducing their oral health-related morbidity and disability and improving their well-being and development* [104]. Its strategic approach is to improve the competency of the oral health workforce engaged in the delivery of services in the public health sectors in Nigeria, while ensuring the private sector and non-governmental organizations complement health care delivery at the national, state and local government levels. Second, it promotes integrated oral health care as a cost-effective way to reduce the oral disease burden. Third, efforts will be made to increase funding and expand the fiscal space for oral health care, as oral health diseases disproportionally affect the poor and socially disadvantaged members of society, and out-of-pocket expenditures for oral diseases may have catastrophic effects on households in Nigeria [105]. Finally, the government also promotes the conduct of oral health research, and the monitoring and evaluation of the oral health policy and national oral health programs to ensure accountability and learning.

The policy thrust for ECC prevention is to integrate oral health care into health care for mothers and pre-school children health care. The strategic direction is to promote the access to routine oral care for pregnant women; promote children's visits to the dentist by their first birthday; ensure access and use of fluoridated toothpaste daily by pre-school children; ensure pre-school children brush their teeth twice daily; and support efforts that ensure preschool children do not consume free sugar in-between-meals more than once a day. In addition, the building of healthful settings that support oral health care will be promoted, such as ensuring schools have policies on reduced access to sugary snacks within and around school premises and ensuring that childcare givers can correctly decipher the caries status of children will be promoted. In addition, the policy promotes structural changes that support enforcement of regulation on fluoridation of all toothpastes in Nigeria; institution and enforcement of regulations on food labeling for sugar content, reduction of sugar in foods; marketing and promotion of sugary foods to children; taxes on sugar-sweetened beverages; and the provision of oral health care as part of the maternal and child health care package in primary health centers [104].

The national strategic approach to ECC management in Nigeria is primary prevention: Screening for diseases and referral for treatment is a primary prevention strategy. However, the linkage to care following oral health screening has been very poor in Nigeria [106]. Linkage to care may be improved through community-based oral health care service delivery. The number of dental care centers in 2013/14—is limited and concentrated in urban Nigeria. There were only 679 public and private dental clinics for the population of over 180 million in Nigeria [107]. Distance to oral health clinics is a challenge for caries management in children in Nigeria [107]. Running mobile clinics would help to address this challenge [108].

In addition to physical barrier, there is also financial barrier to care access in Nigeria. Over 80% of dental caries in the primary dentition remain untreated [109]. Universal health coverage programs can help reduce the burden of health care borne by individuals. However, <15% of the population in Nigeria have access to health insurance with over 80% of Nigerians paying out-of-pocket for health care [105]. Also, the use of conditional cash transfer may improve the uptake of oral health care services [110] as this strategy improved the uptake of maternal care services by mothers in Nigeria [111]. There is a new national health insurance scheme policy that makes it compulsory for all Nigerians to have national health insurance. It is important to evaluate how this scheme may improve the access of children with early childhood caries to preventive and curative care services.

Planning for ECC control and management is also limited in the absence of data [6]: Nigeria does not have representative national oral health data on the prevalence of ECC and individual-, family-, community-, and governance-related factors that increases the risk for ECC, or on the barriers and challenges to preventive and curative dental service utilization by pre-school children. Data on the prevalence of ECC can be collected as part of the National Demographic Health Survey thereby ensuring a regular and cost-effective data collection process. The availability of regular nationally representative data on ECC will help facilitate the development and implementation of a national oral health program, and its monitoring.

#### Thailand

The recent Thailand National Oral Health Survey found a high ECC prevalence in both 3- and 5-year-old children (52 and 76%, respectively). The survey in 2017 revealed a lower caries prevalence among 5-year-old children, compared to the previous survey in 2006 (75.6% in 2017 vs. 80.6% in 2006). However, most cases of ECC remained untreated [26]. The Bureau of Dental Health, Department of Health, Ministry of Public Health is responsible for improving oral health and has been focusing on young children in the past decades. Due to a shortage of oral health manpower in Thailand, the Bureau of Dental Health focuses on the primary prevention of dental caries, especially the reduction of sugar consumption through the “Sweet Enough Network” [112]. This program, which started in 2002 led to a national policy to stop sugar being added to infant formulas in 2006 and is believed to be a key explanation for the lower rate of ECC in 3-years-old children in the last two National Oral Health Surveys. Recently, the Ministry of Public Health launched another campaign, “Tooth-brushing on the first tooth eruption with appropriate fluoride toothpaste.” This campaign aims to encourage community participation and provide hands-on training for the mothers or caregivers on how to detect and remove dental plaque by brushing the baby's first teeth to erupt from around 6 months of age [113]. The aim is to promote oral health self-care for children and their families through dental therapists and trained village health volunteers country-wide. Dental therapists also provide oral health examinations and fluoride varnish applications at day care centers as secondary prevention for ECC. In the past decade, dental therapists and public health dentists have received hands-on training in ECC diagnosis [114] and management using SMART (Simplified Modified Atraumatic Restorative Treatment) as tertiary prevention for open dentine cavities especially in primary molars. SMART is based on partial caries removal, selective removal of soft dentine with spoon excavators and fillings with capsulated glass ionomer cements; the technique is minimally-invasive, painless and acceptable to young children [115]. Recently, in some provinces 38% Sliver Diamine Fluoride has been used for secondary or even tertiary prevention of ECC. Even though Thailand has dental therapists distributed country-wide, they are overloaded with the responsibility of providing simple treatment and promoting oral health for all age groups including pre-school children, school children, adults and the elderly at sub-district health centers. It would be advantageous in the future to have someone solely focused on and responsible for ECC management in the community in order to reduce ECC prevalence and contribute to a better quality of life for pre-school children. How to retain long-term community dental therapists responsible for non-invasive ECC interventions as part of ECC management under the supervision of a public health dentist is a challenge.

#### United Kingdom (UK)

The prevalence rate of ECC for 5-year-old children in UK was around 23%. The nations of the UK have similar rates (with slight differences). The overall prevalence figure given is for England only but is considered broadly representative as there is no reliable combined data for the whole UK [27]. Almost all of child dental health care in the UK is carried out under the National Health Service (NHS) and government-funded, so free at point of care. England, and the devolved nations (Scotland, Wales, and Northern Ireland) have health, and oral health, as a decentralized responsibility. Each nation has taken a different approach to looking after children's teeth in community oral health programs/campaigns and dental care remuneration systems. National oral health surveys have been carried out regularly since the 1980s and give a picture for children aged around 5-, 8-, and 12-years old (the beginning, middle and end of primary school). They show continuing widespread variation in the prevalence and severity of experience of dental caries [116], extending across geographical areas, deprivation and ethnic groups. The monitoring programs not only allowed scrutiny of the oral health of children in general but also an opportunity to assess the impact of oral health promotion programs such as ChildSmile [117] in Scotland and Designed to Smile in Wales [118].

Dental therapist/ hygienists and dental hygienists have a limited scope of practice but are trained to the same standard as dentists in providing these. However, despite the financial benefits of them providing aspects of care, they have not been successfully incorporated into the workforce for a number of reasons [119]. There are very few specialists in pediatric dentistry and these are concentrated mainly in urban areas or are consultants who manage complex care needs such as children who have craniofacial abnormalities, hypodontia or are medically compromised [120].

The large oral health surveillance programs are carried out every 10-years (for England, Wales, and Northern Ireland), with the last one in 2013. In Scotland, they are every 2-years for 5-year-olds, with the last one in 2018.

In 2013, a lower proportion of 5-year old children had obvious decay experience in primary teeth in England (31%, dmft 0.9) than children in Wales (41%, 1.5), Northern Ireland (40%, 1.4), and at that time, in Scotland [116]. However, the smaller, more frequent and more recent national programs show some improvement in those nations.

##### England

The proportion of children with visible caries in 2008 was 31% (the dmft at that time was 1.1) and has dropped to 28% in 2012 (0.9), 25% in 2015, and 23% in 2017 (0.8), remaining there in 2019 [27]. In the most deprived groups, 35% of children experienced caries, compared to 14% in the most affluent group. There was also higher experience in Black, Asian and Minority Ethnic groups [27].

##### Scotland

The proportion of 5-year-olds with visible caries experience has shown a progressive reduction from 60% in 1988 (dmft 2.8) to 55% in 2003, 42% in 2008, 36% in 2010, 33% in 2012, 32% in 2014, 31% in 2016, and 29% in 2018 (dmft 1.1) [121]. The change from a flatline to an upward trajectory, however, did not happen until around 2005/6 and was associated with the Childsmile program [117]. Although there has been a reduction in caries experience for children across all socio-economic groups, the inequality gap has remained constant at around 30% difference in decay experience for children in the lowest socioeconomic level (SE) groups compared to those in the highest, i.e., around 14% of children in the most affluent fifth of the population experience visible caries, compared to 44% for those children in the most deprived fifth of society [121].

##### Wales

In 2009, around 50% of 5-year-olds in Wales had visible caries, a figure that had remained static for the preceeding 10-years (dmft was 1.98). Similar to Scotland, Wales brought in a program funded by the Welsh Government called Designed to Smile but in Wales, the program was specifically targeted toward children in areas with the highest caries prevalence [122]. In 2013 (the last survey), this had dropped to around 41% (dmft 1.5) [116].

In slight contrast to Scotland, and perhaps linked to the targeted design of the program, the most deprived fifth of children have seen the largest reduction of 15% in decay prevalence against an average of 13% across all groups (Welsh Government 2019). In 2015/16 the dmft had dropped to 1.22 [122].

Overall, the future direction of policy seems set to be determined by the devolved countries own health systems for Scotland, Wales and Northern Ireland. There are common moves to try to shift the focus toward prevention both outside the dental surgery and inside. However, moving away from payment systems that incentivize “treatment” or even “no treatment” rather than delivery of high-quality preventive care and behavior change is elusive. There is a significant movement from the Welsh government to move toward a prevention-based payment system. This is currently being implemented. The role of the Soft Drinks Industry Levy [123] or “sugar tax” on dental caries, and how much, if any, they benefit those children in the most deprived groups, is unclear.

#### United States of America

The 2016 national survey reported that 21% 2- to 5-year-old children in USA had ECC [28]. Public health measures like water fluoridation and the widespread use of fluoride toothpaste instituted and promoted in the United States (US) since the 1960s, have resulted in a decline in prevalence of caries in children and the whole population. In conjunction with other national programs, there have been some degree of success: Data from the National Health and Nutrition Examination Survey (NHANES) 2015–2016 indicate that prevalence of dental caries and untreated dental caries in pre-school children have shown a slight decrease since 2011. However, it is still a fact that total dental caries increases with age, going from 21.4% among children 2–5 to 50.5% among those aged 6–11 [28]. Furthermore, the improvement in oral health has not been consistent for all ages or socio-economic groups. For both total and untreated caries, prevalence decreased as family income level increased. When compared to other population groups in the US, American Indian, Alaska Native (AI/AN) preschool children have the highest level of tooth decay (71.3%), more than 4 times higher than white non-Hispanic children (24.9%), followed by Mexican American children (41.5%), and non-Hispanic black children (30.3%) [124]. Caries prevalence in these ethnic minorities is reportedly due to differences in host, bacterial, behavioral, sociodemographic and environmental risk factors [28, 124].

Government funded programs like Medicaid and Children's Health Insurance Program (CHIP) offer comprehensive dental coverage for families below the poverty level who fall within vulnerable populations at risk for dental decay. In addition to restorative care (tertiary prevention), these programs cover minimally invasive treatments aligning with important primary prevention policies brought forward by American Dental Association and American Association of Pediatric Dentistry. These include the establishment of a dental home when the first tooth erupts or at age 1, which encompasses all preventive measures consistent with a dental home, as well as secondary prevention programs, like sealant programs in schools. Other government-funded programs like Headstart (HS) and Women, Infants and Children (WIC) provide education, screenings, check-ups, nutrition services and referrals. Sadly, utilization of these programs is not as high as it should be, due to different barriers. In the past, efforts to reduce ECC have focused more on providers and funding rather than considering social determinants of health of families and behavioral issues, resulting in mixed success [125].

A recent study found that, all else being equal, the odds of untreated dental caries associated with non-financial barriers were higher than the odds of untreated caries associated with financial barriers for children, perhaps thanks to comprehensive dental coverage through Medicaid and CHIP [126]. Additionally, factors such as a lack of convenient appointment times, parental busyness, prohibitive distance to a dentist's office or a child's fear of the dentist play a critical role in untreated caries among children and deserve more attention. There is a need for alternative interventions such as school-based prevention and sealant programs, which overcome many of the cited barriers [126]. But just as important, education for parents to raise their oral health literacy is imperative for them to learn the value of utilization of available preventive resources and establishing oral health behaviors that will positively impact the child's life course [127].

The biggest challenge of managing ECC in the US, as 80% of the burden of disease falls into 20% of the population, is to implement evidence-based strategies to overcome the social determinants of health so vulnerable children have a chance for improved, sustained oral health.

#### Venezuela

In Venezuela, government health policies focus on community-based health programs to promote overall well-being, prioritizing primary prevention. General measures such as oral health education and the use of fluoridated toothpaste are advised for school settings [128]. No concise information regarding specific measures to prevent or treat ECC is available, and no ECC programs are currently being implemented in the country [129, 130].

There is a scarcity of information on the epidemiology, etiology and effective prevention of ECC in preschool children in Venezuela. Most data have been collected by university researchers with diverse methodologies and small population samples, which do not allow for a national mapping of the ECC situation in the country at this time. Based on two studies in 1995 and 2008, the prevalence of ECC in Venezuelan children up to age 5 varied from 24 to 74% [131, 132].

Population primary prevention measures have included salt fluoridation since 1997 (200–220 milligram potassium fluoride per salt kilogram) [133]. Secondary preventive measures and ECC management are not addressed in national health programs.

A large segment of Venezuelan infants and preschoolers are at risk of ECC and severe early childhood caries (SECC), based on the reported malnutrition and poverty levels for the county [132, 134]. A 2007 study showed that any children of lower socioeconomic status were not practicing any healthy oral care habits at all [135].

Regrettably, at the present time ECC is not a priority for national health programs. The main challenge remains the acknowledgment of ECC as a public health problem and the development of evidence-based dental public health policies to address the ECC epidemic in a sustainable way.

## Discussion

The FDI World Dental Federation has called for action to control the high global prevalence of ECC and encourage the use of preventive dental medicine to reduce the burden of tooth decay [136]. The dental experts of the World Health Organization (WHO) Global Consultation reviewed the risk factors of ECC and recommended that “national health authorities should develop strategies and implement interventions aimed at preventing and controlling ECC” [137]. The present review reported the following highlights. First, the prevalence of ECC varies across the globe. The burden of ECC remains high in most of the developing countries (Table 1). The distribution of ECC is disproportional as children from low-income countries have higher ECC prevalence rates and higher mean dmft scores. Apart from that, the dentist to population ratios and the pediatric dentist to children-under-age-5 ratios are small in developing or low-income countries. This reveals that an insufficient number of dental care professionals is a common problem in these countries, which may have implications for ECC control (Table 2). Furthermore, different patterns to manage, finance, and deliver child oral health care services were found among the countries (Table 3). Most of the countries in this review provide oral health coverage for young children. However, most of the preventive strategies focus on the individual level and less on the macro-level.

The difference in the prevalence of ECC between countries/regions may be attributed to different oral health care provisions which may be affected by a wide range of social, economic and political factors, as well as the use of fluoride and oral health related habits. Also, there were different caries diagnosis criteria used—not all used the WHO criteria (such as Cambodia, India, and UK)and not all sampling was similar. This review found that countries that provided free oral health assessments (dental checkups) and comprehensive public health programs such as Japan, UK, and USA reported a lower prevalence of ECC. Countries without prevention programs like Cambodia and India showed a higher prevalence of ECC. Macro-level interventions for ECC management such as water fluoridation, salt fluoridation and sugar taxation were only reported by some countries/regions (Australia, Brazil, Hong Kong, Thailand, UK, USA, and Venezuela). But the prevalence of ECC among 5-year-old children in these countries/regions varied (from 23 to 80%). More evidence is needed to investigate the factors that influence the success of these macro-level interventions in reducing the prevalence of ECC and additionally the effects of different countries' contexts (culture, politics, dental care provision).

Though oral health care provision plays a role in managing ECC, there was no government-subsidized dental care service for preschool children reported for many of the countries/regions included, namely Cambodia, India, Venezuela, and Hong Kong. ECC management may not yet be regarded as a priority in these countries even though the burden of ECC for them is enormous. Universal oral health coverage for young children will not transpire if nations do not place greater value on child oral health. However, in countries with universal coverage for young children, such as Thailand and Indonesia, there are still barriers to accessing dental treatment. From Table 2, the number of dentists, pediatric dentists and resources available is not adequate to accommodate the actual treatment needs of children with ECC. Even if there are a high number of pediatric dentists, most of them usually work in the private sector in the capital or big cities. Although dental care is a part of universal health coverage in some countries, there is limited information regarding the number or the percentage of young children in each country who had access to dental services. The distribution of ECC is disproportional as higher prevalence of ECC was found among underprivileged children who come from rural and remote areas [138]. Therefore, sustaining effective child oral health programs especially tertiary oral health care prevention in rural and remote areas is a challenge. Any programs or policies providing incentives for dentists to provide dental care in government-funded facilities or private sectors should be encouraged.

The medical and dental interface is pivotal. Basic oral health care services need to be integrated into the medical children's care. Training and engagement of general health care workers to provide oral health care for children show promise in reducing child oral health disparities [139]. Allied medical or health personnel, like midwives, nurses, community health extension workers and primary care providers, have great potential for screening oral health conditions, offering guidance on caries prevention for parents and providing simple preventive measures [140]. Also, kindergarten teachers may play a pivotal role in reducing ECC disparities, as proposed by some countries/regions such as Hong Kong [14]. Any initiatives or programs providing incentives for dental professionals to collaborate with other categories of healthcare workers and schoolteachers should be encouraged and expanded at the local or national levels. In addition to address child oral health as an integral part of primary health care and childhood education, the engagement of the so-called “midlevel providers” such as dental hygienists or dental therapists who have a scope of practice limited to certain procedures or activities in the workforce can bridge some of the access gaps. Nevertheless, each country has its regulations regarding the scope of midlevel providers in the clinic and community. Innovative workforce arrangement that includes task-shifting and task-sharing is needed to improve child's oral health, particularly for those underserved populations with higher rates of disease prevalence. More data on medical and dental interface in each country and the system should be discussed.

The proportion of the available dental workforce and disease prevalence seems to make it unrealistic to suggest providing surgical or conventional restorative treatment to millions of children suffering from ECC in such countries. New intervention protocols that are highly effective, easy to deliver and require fewer sensitive techniques and dental equipment need to be developed and adopted especially in the countries with a high ECC prevalence and low dentist/population ratio. Minimally invasive approaches such as Atraumatic Restorative Treatment, the Hall Technique and silver diamine fluoride application that can be performed in field settings or using fewer resources by trained healthcare workers would be another possible solution [141]. Innovative dental education should move toward the incorporation of non-surgical approaches as essential elements of a comprehensive ECC management plan [142].

The present review has a few limitations. First, the number of countries included in the review is limited and does not represent the global profile—the countries are a convenience sample of interested collaborators who were purposefully invited and who agreed to participate in this review. Also, due to the limitation of narrative review with no predefined search strategy, potential bias from searching and selecting data could occur. The collection and gathering of the data differed from countries to countries. A systematic review with more rigorous search and selection process is required. Besides, not all the data on the prevalence of ECC were nationally representative data. The impact of national preventive programs is not fully understood without adequate research on assessing oral health policies. This limits the ability of countries to design evidence-informed policies and programs for ECC control. Despites these, the current critical review contributed by a large group of dental experts could be an initial source of evidence that could provide experts' intuitive and explicit perspectives about oral health policies in managing ECC. Regular national oral health surveys should be conducted and a concerted campaign should be launched to drive the development of a global agenda to prevent ECC and manage it when prevention fails. Well-designed oral health policies that effectively address the structural determinants of ECC are needed. Research on implementation science and translation of research into practice is also essential to understand the feasibility and effectiveness of existing and planned preventive strategies.

To conclude, ECC remains a global health challenge with a high societal and financial burden, despite being a largely preventable disease. The dental workforce is limited in most countries and is likely to continue to be so. National or regional preventive programs to tackle ECC are not yet prioritized in many countries and evidence to support demonstration projects or policies is limited. Further research on the cost-effectiveness of interventions and demonstration projects is required to provide information for policymakers. A global campaign for the control and elimination of ECC is long overdue for a disease that has such huge public health implications.

## Author Contributions

All authors collected the data in different countries/regions and drafted and reviewed the manuscript.

## Conflict of Interest

The authors declare that the research was conducted in the absence of any commercial or financial relationships that could be construed as a potential conflict of interest.
